# Single-cell RNAseq reveals adverse metabolic transcriptional program in intrahepatic cholangiocarcinoma malignant cells

**DOI:** 10.1016/j.bbrep.2025.101949

**Published:** 2025-02-15

**Authors:** Christophe Desterke, Raquel Francés, Claudia Monge, Yuanji Fu, Agnès Marchio, Pascal Pineau, Jorge Mata-Garrido

**Affiliations:** a Faculté de Médecine du Kremlin Bicêtre, Université Paris-Saclay, INSERM UMRS-1310, Le Kremlin-Bicêtre, France; b Energy & Memory, Brain Plasticity Unit, CNRS, ESPCI Paris, PSL Research University, Paris, France; c Institut Pasteur, Université Paris Cité, Unité Organisation Nucléaire et Oncogenèse, INSERM U993, Paris, France; d Université Paris Cité, INSERM, CNRS, Institut Necker Enfants Malades, F-75015, Paris, France

**Keywords:** Intrahepatic cholangiocarcinoma, Metabolic score, Enzyme, Single cell RNA sequencing, Liver cancer, Keggmetascore

## Abstract

Intrahepatic cholangiocarcinoma (ICA) is a highly aggressive primary liver cancer, which originates from the epithelial cells of the bile ducts. The transcriptional profile of metabolic enzymes was investigated at both bulk and single-cell levels in tumor samples from distinct ICA cohorts. In a training cohort (TCGA consortium), 16 genes encoding for metabolic enzymes were found overexpressed in cases with poor survival. A computed metabolic gene expression score was significantly associated with worse ICA prognosis at the univariate level (overall survival [OS] log-rank p = 8.2e-4). After adjusting for Ishak fibrosis score and tumor staging, the metabolic expression remained an independent predictor of poor prognosis (multivariate OS log-rank p = 0.01). Seven genes encoding key enzymes (FH, MAT2B, PLOD2, PLOD1, PDE6D, ALDOC, and NT5DC3) were validated as markers of the proliferative subclass of ICA in the GSE32225 dataset, related to poor prognosis. The metabolic score was significantly different between the inflammatory and proliferative subclasses in the validation cohort (p < 2.2e-16). At the single-cell level, in the tumor microenvironment of 10 ICA patients, these seven enzymes were predominantly expressed by malignant cells. The single-cell metabolic score was thus higher in malignant cells. This study identifies a metabolic transcriptional program linked to poor prognosis in ICA, independent of fibrosis and tumor staging.

## Highlights

• **Metabolic transcriptional program** linked to poor prognosis identified in intrahepatic cholangiocarcinoma (ICA).
• **16 metabolic enzymes** were overexpressed in ICA patients with poor survival in the TCGA cohort.
• A metabolic gene score predicts worse OS, independent of fibrosis and tumor stage.
• Seven metabolic genes mark the proliferative ICA subclass.
• Single-cell analysis shows these enzymes are mainly in malignant tumor cells.
• The metabolic gene expression score was significantly higher in **proliferative ICA subclass**, linking it to poor prognosis.

## 1 Introduction

Liver cancer is a leading cause of cancer-related mortality worldwide, with its two major subtypes, hepatocellular carcinoma (HCC) and intrahepatic cholangiocarcinoma (ICA), posing significant clinical challenges [1,2]. HCC arises from hepatocytes [3], while ICA arises from the epithelial cells of the intrahepatic and extrahepatic bile ducts and occurs proximal to the segmental biliary ducts [4,5]. Though less common than HCC, ICA is particularly aggressive, often diagnosed at advanced stages and associated with poor survival outcomes [6]. The prognosis for ICA remains bleak due to its high recurrence rates, limited treatment options, and frequent resistance to conventional therapies such as chemotherapy, radiation, and surgical resection [6]. Despite advances in diagnostic techniques and therapeutic strategies, the survival rates for ICA have not significantly improved, necessitating further exploration of the molecular mechanisms driving this malignancy.

One area of growing interest in cancer research is the role of metabolic reprogramming, a hallmark of cancer, in driving tumor initiation, progression, and resistance to therapy [7]. Tumor cells, including those in liver cancers, undergo significant alterations in their metabolism to support rapid cell growth, evade immune surveillance, and adapt to the often-harsh microenvironment of the tumor. These changes involve the upregulation of key metabolic pathways such as glycolysis, glutaminolysis, and lipid metabolism, which enable tumor cells to meet their increased energy and biosynthetic demands [7]. Metabolic dysregulation has been extensively studied in HCC, where network rewiring, such as the Warburg effect and increased lipid metabolism, are recognized as critical to tumor progression [[8], [9], [10]]. At genomic level, mutations of IDH are mainly observed in iCCA and are associated with poor differentiation of hepatic progenitor cells resulting from production of the oncometabolite D-2-HydroxyGlutarate [11,12]. Altered lipid metabolism is a prominent metabolic modification in cancer. Reactivation of de novo lipogenesis and increased of fatty acid synthesis in cancer making them independent of exogenous lipid uptake [13]. During iCCA, MAL2 is highly expressed in ICC and maintains EGFR's membrane localization. Its actin subsequently activates the PI3K/AKT/SREBP-1 signaling pathway, leading the upregulation of key fatty acid synthesis genes such as FASN and SCD, thereby promoting lipid accumulation [14]. It is interesting to investigated deeply metabolism pathways especially at transcriptional level during iCCA. The identification of these metabolic enzymes and their associated pathways is crucial, as they could serve as potential biomarkers for prognosis or targets for novel therapies. Moreover, understanding the specific metabolic changes that occur in ICA, both at the bulk tumor and single-cell levels, could offer insights into tumor heterogeneity and the diverse responses to treatment observed in clinical practice.

In this study, we investigate the transcriptional landscape of metabolic enzymes in ICA by analyzing RNA-sequencing data from bulk and single-cell tumor samples. Our goal is to identify genes encoding for metabolic enzymes that are differentially expressed in ICA and associated with adverse clinical outcomes. We developped a specific metabolism R-package: “keggmetascore” based on 41 KEGG metabolism pathways and single sample scoring at transcriptomic or proteomic levels. By focusing on metabolism, we aim to uncover new insights into ICA biology, with particular attention to the role of metabolic reprogramming in tumor progression and prognosis. We hypothesize that certain metabolic transcriptional programs may serve as drivers of ICA aggressiveness and that these programs are primarily active in malignant cells within the tumor microenvironment. By characterizing these metabolic signatures, we hope to pave the way for future research aimed at targeting metabolic vulnerabilities in ICA and improving therapeutic outcomes for patients with this devastating cancer.

## 2 Material and methods

### 2.1 Transcriptome datasets of intrahepatic cholangiocarcinoma tumors

#### 2.1.1 Training cohort of RNA-sequencing

Training cohort data (n = 29) from the TCGA cholangiocarcinoma study [15] was obtained from the cBioPortal platform at https://www.cbioportal.org/study/summary?id=chol_tcga (accessed on September 27th, 2024). For transcriptome analysis, RNA-sequencing data normalized using the RSEM pipeline was selected and scaled using a logarithmic transformation (log2 + 1) [16]. The normalized transcriptome data was integrated with corresponding clinical information, and samples were filtered to include only the intrahepatic cholangiocarcinoma (ICA) subclass (n = 29, Table 1). For this cohort, the performance status of ECOG (Eastern Cooperative Oncology Group) was evaluated: majority of patients were from score 0 (58 percents), followed by patients of score 1(37 percents, Table 1), also Ishak fibrosis score was quantified: majority of patients were found without fibrosis (59 percents), on third of them were found with a portal fibrosis, and two patients were detected with Fibrous speta (Table 1). Evaluation of tumor grade for these patients identified 1 patient of grade 4, 15 patients of grade 3, and 13 patients of grade 1 (Table 1). Majority of patients presented no nodes invasion (76 percents, Table 1) and no metastasis (86 percents, Table 1). Tumor staging of AJCC comitee characterized heterogenous stages for patients composing this cohort: 17 patients of stage I, 9 patients of stage II, 2 patients of stage IVA, and 1 patient of stage IVB (Table 1). A minority of tumors presented micro-vascular invasion (n = 4) and perineural invasion (n = 4) (Table 1).

**Table 1:** Clinical characteristics of TCGA intrahepatic cholangiocarcinoma stratified on metabolism categories low and high scores (n = 29).

Variable	Level	low (n = 8)	high (n = 21)	Total (n = 29)	p-value
ECOG SCORE	0	4 (50.0)	10 (62.5)	14 (58.3)	
	1	4 (50.0)	5 (31.2)	9 (37.5)	
	3	0 (0.0)	1 (6.2)	1 (4.2)	0.5647181
ISHAK FIBROSIS SCORE	1,2 - Portal Fibrosis	3 (42.9)	4 (26.7)	7 (31.8)	
	3,4 - Fibrous Speta	0 (0.0)	2 (13.3)	2 (9.1)	
	0 - No Fibrosis	4 (57.1)	9 (60.0)	13 (59.1)	0.5134709
GRADE	G2	3 (37.5)	10 (47.6)	13 (44.8)	
	G3	5 (62.5)	10 (47.6)	15 (51.7)	
	G4	0 (0.0)	1 (4.8)	1 (3.4)	0.6834190
AJCC TUMOR PATHOLOGIC PT	T2b	0 (0.0)	3 (14.3)	3 (10.3)	
	T2	0 (0.0)	5 (23.8)	5 (17.2)	
	T2a	1 (12.5)	1 (4.8)	2 (6.9)	
	T1	6 (75.0)	11 (52.4)	17 (58.6)	
	T3	1 (12.5)	1 (4.8)	2 (6.9)	0.3355886
AJCC NODES PATHOLOGIC PN	NX	0 (0.0)	4 (19.0)	4 (13.8)	
	N0	7 (87.5)	15 (71.4)	22 (75.9)	
	N1	1 (12.5)	2 (9.5)	3 (10.3)	0.4125795
AJCC METASTASIS PATHOLOGIC PM	M0	7 (87.5)	18 (85.7)	25 (86.2)	
	MX	0 (0.0)	3 (14.3)	3 (10.3)	
	M1	1 (12.5)	0 (0.0)	1 (3.4)	0.1518291
AJCC PATHOLOGIC TUMOR STAGE	Stage II	1 (12.5)	8 (38.1)	9 (31.0)	
	Stage I	6 (75.0)	11 (52.4)	17 (58.6)	
	Stage IVA	0 (0.0)	2 (9.5)	2 (6.9)	
	Stage IVB	1 (12.5)	0 (0.0)	1 (3.4)	0.1635401
VASCULAR INVASION	None	7 (87.5)	17 (85.0)	24 (85.7)	
	Micro	1 (12.5)	3 (15.0)	4 (14.3)	1.0000000
PERINEURAL INVASION	YES	0 (0.0)	4 (22.2)	4 (15.4)	
	NO	8 (100.0)	14 (77.8)	22 (84.6)	0.3894405
TUMOR SITE	Bile duct	8 (100.0)	21 (100.0)	29 (100.0)	0.0157768
AFP AT PROCUREMENT	mean (sd)	2.5 (0.6)	3.8 (1.8)	3.4 (1.6)	0.0532344
OS STATUS	0	7 (87.5)	8 (38.1)	15 (51.7)	
	1	1 (12.5)	13 (61.9)	14 (48.3)	0.0495386
OS MONTHS	mean (sd)	36.8 (20.9)	13.9 (12.4)	20.7 (18.3)	0.0003678

#### 2.1.2 Validation cohort of transcriptome

Validation cohort was normalized transcriptome data from the GSE32225 dataset [17] was processed using the GEOquery R package version 2.70.0 [18]. The data was annotated according to the provided technological platform, GPL8432, corresponding to the Illumina HumanRef-8 WG-DASL v3.0 microarray. This corhort of transcriptome is composed of 149 iCCA tumor experiments. Among these samples, a minority (n = 57, 38 percents) were classified as “inflammation” because overexpressing a pletore of interleukins and chemokines (IL3, IL4, IL6, IL10, IL17A, and CCL19) associated to good prognosis of patients and these tumors were found histologically well differentiated. A majority of tumors composing this cohort (n = 92, 62 percents) were classified as “proliferative” composed of tumors moderate or poorly differentiated in histology, associated to poor prognosis of the patients, and harboring activation of oncogenic pathaways including RS/MAPK kinase and MET with presence of BRAF and KRAS mutations.

### 2.2 Single cell transcriptome of intrahepatic cholangiocarcinoma tumors

Individual batch of 10x Genomics experiments of sc-RNAseq from dataset GSE125449 [19] were downloaded on Gene Expression Omnibus website [20]. After batch integration with canonical correlation, sc-RNAseq experiments were filtrated for analyzing only the 6033 cells of ICA samples from 10 distinct patients. Downstream single cell RNA-sequencing analyses were followed in Seurat R-package version 5.1.0 [21].

### 2.3 Mammalian metabolic transcriptional program

Mammalian Metabolic Enzyme Database [22], was downloaded at the following web address: https://esbl.nhlbi.nih.gov/Databases/KSBP2/Targets/Lists/MetabolicEnzymes/MetabolicEnzymeDatabase.html (accessed on 2024, September 27th) and annotated with Ensembl Biomart database version 112 [23] available at the following web address: http://mart.ensembl.org/info/data/biomart/index.html (accessed on 2024, September 27th).

### 2.4 Transcriptome analyses

Transcriptome analyses were performed in R software environment version 4.3.3. Differential expression analysis was performed with transpipe14 R-package which implement limma algorithm [24] and available at the address: https://github.com/cdesterke/transpipe14 (accessed on 2024, September 23rd) [25]. Expression heatmap and unsupervized clustering (Euclidean distances and Ward.D2 methods) were done with support of pheatmap R-package version 1.0.12.

### 2.5 Survival analyses

The expression of mammalian enzymes was individually tested for overall survival in ICA patients using univariate analyses, implemented through the R-package loopcolcox version 1.0.0, available at: https://github.com/cdesterke/loopcolcox (accessed on September 23, 2024) [25]. Univariate log-rank tests and Kaplan-Meier plots of the metabolic score were generated using the survminer R-package version 0.4.9 [26]. A multivariate Cox overall survival model was constructed with the survival package, incorporating covariates such as metabolic expression score, Ishak fibrosis score [27], and tumor staging. Linearity of Cox residuals for each covariate was evaluated through individual and global Schoenfeld tests [28]. A forest plot for the overall survival model was generated using the GGally R-package version 2.2.1, and model statistics were exported using the broom R-package version 1.0.6. Bootstrapping of the multivariate overall survival model was conducted with the rms R-package version 6.8–1, performing 500 iterations at a failure time of 18 months [29]. A nomogram at the validated failure time was created using the regplot R-package version 1.1.

### 2.6 Binomial analyses on up-regulated adverse enzymes

The individual expression of adverse enzymes was evaluated based on the binomial outcome of ICA proliferation subclass status in the validation cohort GSE32225. This was done by performing iterative logit analyses using the logitloop R-package version 1.0.0, available at: https://github.com/cdesterke/logitloop (accessed on September 23, 2024). Significant enzymes with positive predictive value were further validated through individual ROC curve analyses using the multirocauc R-package version 1.0.0, available at: https://github.com/cdesterke/multirocauc (accessed on September 23, 2024) [30].

### 2.7 Metabolic score

In context of Cox or binomial analyses for bulk transcriptome of ICA tumors, a metabolic expression score was computed with expression of the significant enzymes and concordant enzymes characterized between the two independent cohorts of transcriptome: FH, MAT2B, PLOD2, PLOD1, PDE6D, ALDOC, and NT5DC3. This score computing is based on the following formula (1):

(1) metabolic.score = Σ expression x coefficient

where “expression” represents expression of each enzyme and “coefficient” represent beta coefficient of each enzyme during the prediction. Metabolic score in validation cohort according binomial oucome of “proliferative”status, was computed with collection of logit beta coefficients in the formula instead Cox beta coefficients. Optimal threshold of metabolic score according “proliferation” tumor status on validation cohort GSE32225 was determined with cutpointr R-package version 1.1.2 [31]. Mosaicplot and chisquare between inflammation/proliferation classification and metabolism score categories (low and HIGH) were investigated with vcd R-package version 1.4–13 [32].

### 2.8 Development of keggmetascore R-package

In order to investigate deeply metabolism deregulation in iCCA tumors, a specific R-package was developped. This package allowed to perform at transcriptomic or proteomic levels, single sample scoring of a large panel of metabolism pathways characterized in human cells. Starting from MsigDb-2024 [33], Kyoto Encyclopedia of Genes and Genomes (KEGG) database [34] was extracted in gmt format. In KEGG subset of genesets, metabolism pathway were extracted from “gmt” file with “grepl” instruction according “METABOLISM” keyword. This process allowed to extract 41 KEGG metabolism genesets which were reformat in “gmt” file format after their selection. The “keggmetascore” R-package is compatible of with human transcriptome matrix with gene symbol names or Ensembl gene identifiers [23] as annotation of with proteome matrix with Swissprot/uniprot identifiers [35] as annotation. Based on GSVA algorithm [36], the four single sample score quantification (ssgsea, zscore, gsva, and plage) were implemented in the package and the results could be visualized as heatmap. Differential genesets analysis between two groups of samples could be perform by linear model for microarray (LIMMA) analysis [24] and results could be visualized as volcanoplot. The “keggmetascore” R-package was developed and built in Rstudio version 24.04.2. The documentation was automatically implemented in the package with roxygen2 R-package version 7.3.2. The “keggmetascore” R-package is available for installation at the address: https://github.com/cdesterke/keggmetascore (accessed on 2025, January 20th). Single sample by ssGSEA computing was applied through keggmetascore R-package on expression matrix of GSE32225 transcriptome dataset [17].

### 2.9 Gene regulatory network

Starting from the validation cohort of transcriptome (GSE32225, n = 149 tumors, Table 1), the most variable genes across the samples were extracted according unsupervised method based on variance of genes across samples. Gene regulatory networks in transcriptome of iCCA tumors were investigated with Co-Expression Network and Connectivity Analyses (WGCNA) on the most variable genes [37,38]. WGCNA R-package, version 1.72–5 was employed to select soft power fixed to twelve for an optimal topological model. This included the construction of an adjacency matrix and module identification based on expression of most variable genes. Cell phenotype–trait correlations were performed for the seven identified gene modules. On positive correlated bleue gene module with metabolism score, a functional enrichment was performed with clusterprofiler R-package version 4.10.1 [39,40], on Gene Ontology Cellular Compartment database (GO:CC) [41].

### 2.10 Single cell RNA-sequencing analyses

Experiments using 10x Genomics sc-RNAseq from the GSE125449 dataset [19] were integrated using the Seurat R-package version 5.1.0 [21] through canonical correlation analysis. After normalizing and scaling the most variable features, dimensionality reduction was conducted via principal component analysis (PCA), followed by t-distributed stochastic neighbor embedding (t-SNE) [42], based on ElbowPlot diagnostics. Visualizations of single-cell data, including dot plots, violin plots, and feature plots, were generated with Seurat. The metabolic score in sc-RNAseq experiments was calculated using the Seurat function "AddModuleScore" using the combined expression of significant and concordant enzymes: FH, MAT2B, PLOD2, PLOD1, PDE6D, ALDOC, and NT5DC3.

### 2.11 Gene-drug interaction detection

With the validated seven activated enzymes in ICA tumors: FH, MAT2B, PLOD2, PLOD1, PDE6D, ALDOC, and NT5DC3, gene-drug interaction detection was performed in the lastest version of Dgldb 5.0 database (https://dgidb.org/downloads,accessed on 2025, January 20th) [43]. Interaction dataset was imported in R software environment and drugs against the seven selected enzymes were filtrated with “grepl” function on regular expression of gene name column. False positive regular expression were removed with filter commande of dplyr R-package version 1.1.4.

## 3 Results

### 3.1 Upregulated metabolic transcriptional program linked to poor prognosis in intrahepatic cholangiocarcinoma

**Fig. 1:**
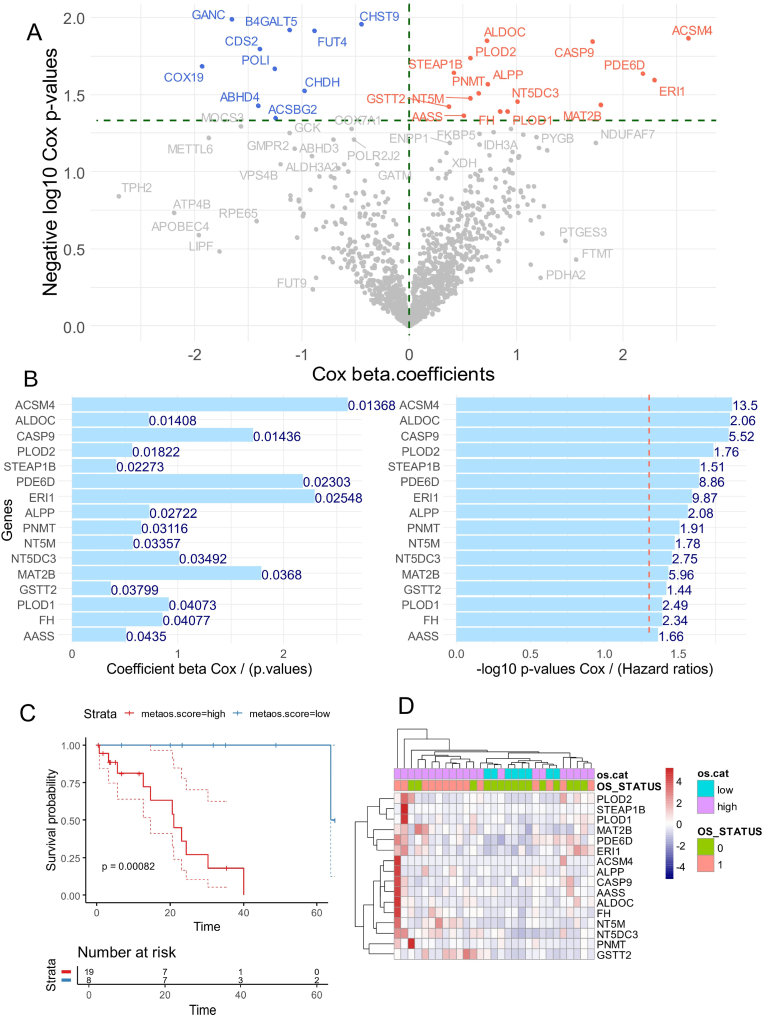
**Adverse expression of metabolic enzymes during intrahepatic cholangiocarcinoma:** TCGA ICA; A/Volcanoplot univariate overall survival Cox analyses against expression of mammalian metabolic enzymes in TCGA-ICA transcriptome; B/Barplot of univariate Cox analyses for the 16 significant enzymes with adverse up regulation in ICA transcriptome; C/Kaplan-Meier and log rank test for overall survival of ICA according low and high categories of computed metabolic score; D/Unsupervised clustering (Euclidean distances) and expression heatmap for the 16 adverse expressed enzymes in ICA transcriptome.

The expression of enzymes listed in the Mammalian Metabolism Database [22] was analyzed for its association with overall survival in 29 patients with intrahepatic cholangiocarcinoma (ICA) from the TCGA-CHOL Firehose cohort (Table 1). Most of the significant enzymes were found to be overexpressed in patients with poor ICA prognosis (Fig. 1A). Sixteen enzymes were identified through univariate Cox analysis as significantly associated with overall survival (Fig. 1B and Supplemental Table 1). These enzymes, ranked by decreasing significance, included: acyl-CoA synthetase medium chain family member 4 (ACSM4), aldolase C (ALDOC), caspase 9 (CASP9), procollagen-lysine,2-oxoglutarate 5-dioxygenase 2 (PLOD2), STEAP family member 1B (STEAP1B), phosphodiesterase 6D (PDE6D), exoribonuclease 1 (ERI1), placental alkaline phosphatase (ALPP), phenylethanolamine N-methyltransferase (PNMT), 5′,3′-nucleotidase, mitochondrial (NT5M), 5′-nucleotidase domain-containing 3 (NT5DC3), methionine adenosyltransferase 2B (MAT2B), glutathione S-transferase theta 2 (GSTT2), procollagen-lysine,2-oxoglutarate 5-dioxygenase 1 (PLOD1), fumarate hydratase (FH), and aminoadipate-semialdehyde synthase (AASS). Based on the expression of these enzymes, a metabolic expression score was calculated, and the optimal threshold for this score was determined based on survival proportion (threshold: 116.17). Stratification by metabolic score identified two groups: eight patients with low scores and 21 with high scores (Table 1). Kaplan-Meier analysis and the log-rank test showed significant differences between these groups (log-rank p-value = 8.2e-4, Fig. 1C). Unsupervised clustering based on the expression of these 16 adverse enzymes (using Euclidean distances) aligned most patients with their clinical phenotypes, including overall survival status (OS_STATUS: 1 = dead, 0 = alive) and metabolic score categories (os.cat: low vs. high) (Fig. 1D).

### 3.2 Metabolism score is an independent adverse parameter in prognosis of intrahepatic cholangiocarcinoma

**Fig. 2:**
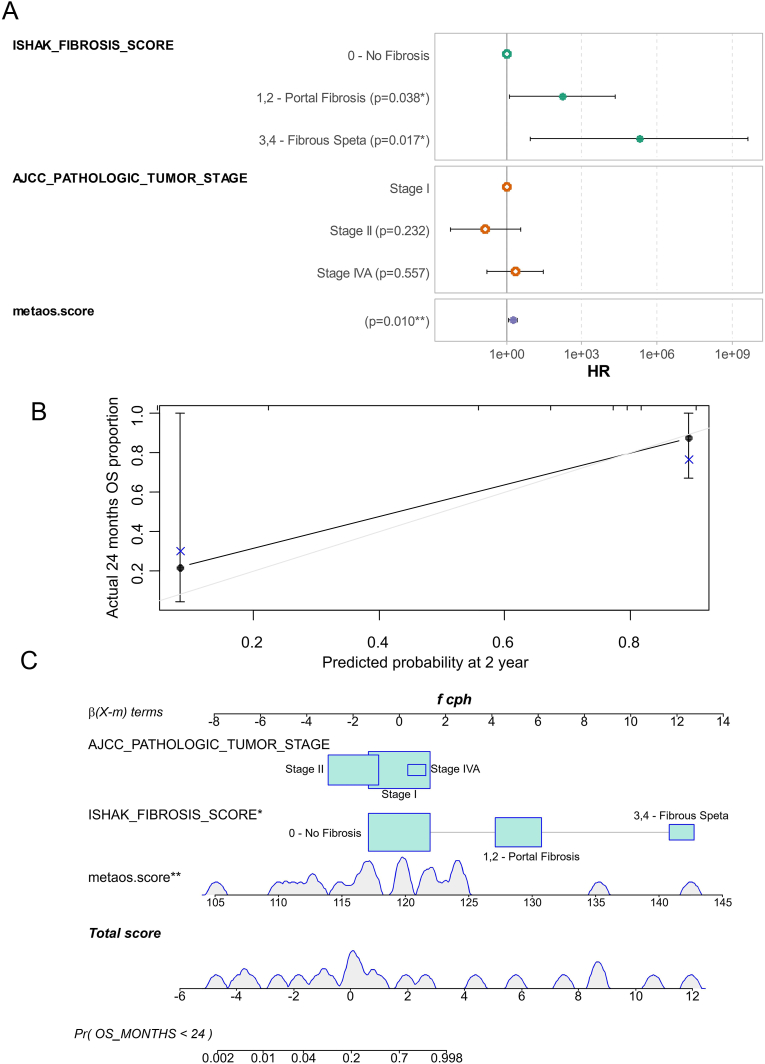
**Metabolic score is an adverse independent parameter in prognosis of intrahepatic cholangiocarcinoma:** A/Forest plot of the overall survival multi-variable model integrating metabolic score, American Joint Committee on Cancer (AJCC) tumor stage and Ishak fibrosis score; B/Bootstrap of the overall survival multi-variable model for prediction of 24-month failure time; C/Nomogram of the multi-variable overall survival model for validated 24 months failure time.

Taking the above as a starting point, and to assess the clinical independence of this expression parameter, a multi-variable overall survival model was constructed, incorporating the metabolic score along with the Ishak fibrosis score and tumor staging. The linearity of the Cox residuals for the covariates was evaluated using both global and individual Schoenfeld tests (Supplemental Fig. 1A). The resulting multi-variable model demonstrated significance with a log-rank p-value of 0.002 and a concordance index of 0.93 (± standard error 0.028) (Fig. 2A). Even after adjusting for the Ishak fibrosis score and tumor staging, the metabolic score remained a significant adverse prognostic factor for ICA (multi-variable p-value = 0.01, Fig. 2A and Table 2). Predictions from the adjusted multi-variable model indicated a progressive increase in death risk with higher metabolic scores (Supplemental Fig. 1B). The multi-variable overall survival model was calibrated using bootstrapping with 500 permutations for a survival time of 24 months (Fig. 2B). The corresponding calibrated nomogram for the 24-month failure time confirmed a wide distribution of points for the metabolic score parameter across the model, with death probability ranging from 0.002 to 0.998 (Fig. 2C) (see Table 3).

**Table 2:** **Keggmetascore of ICA tumor samples from validation cohort GSE32225:** single sample metabolism pathway scores determined by keggmetascore analysis were tested between tumors with low and high metabolism categories, for respective significant pathways: logarithm base 2 fold change (logFC) between High and low categories and corresponding False Discovery Rate (FDR) adjusted p-values were presented as separated columns.

metabolism pathways	logFC	adj.P.Val
PROPANOATE	0.1101	2.26E-21
CYSTEINE_AND_METHIONINE	0.0888	8.00E-18
INOSITOL_PHOSPHATE	0.0646	1.01E-14
PYRUVATE	0.0685	3.98E-14
ARGININE_AND_PROLINE	0.0735	9.48E-13
BETA_ALANINE	0.0813	3.51E-12
BUTANOATE	0.0737	3.66E-11
FATTY_ACID	0.0766	3.63E-10
PURINE	0.0288	4.01E-09
PYRIMIDINE	0.0384	7.35E-08
GLYOXYLATE_AND_DICARBOXYLATE	0.0779	3.83E-07
NICOTINATE_AND_NICOTINAMIDE	−0.0460	8.28E-07
TAURINE_AND_HYPOTAURINE	−0.1162	1.03E-06
PHENYLALANINE	0.0664	1.42E-06
NITROGEN	0.0535	3.72E-06
TRYPTOPHAN	0.0487	7.13E-06
ETHER_LIPID	0.0344	3.71E-05
ARACHIDONIC_ACID	−0.0398	2.45E-04
ALPHA_LINOLENIC_ACID	−0.0552	2.50E-04
FRUCTOSE_AND_MANNOSE	0.0433	1.08E-03
GLUTATHIONE	0.0212	3.55E-03
AMINO_SUGAR_AND_NUCLEOTIDE_SUGAR	0.0246	3.81E-03
GALACTOSE	−0.0293	4.31E-03
GLYCEROPHOSPHOLIPID	−0.0134	3.29E-02

**Table 3:** **Table of multi-variable overall survival model integrating metabolic score, tumor stages and Ishak fibrosis score**: respective columns presented hazard ratios, confident intervals low at 95 percents, confident intervals high at 95 percents, and overall survival multi-variable p-values for each covariate included in the model, control group for ISAK-score fibrosis was no fibrosis, control group AJCC pathological tumor stage was Stage I, meta.score for expression metabolic score was a quantitative parameter.

covariates OS	hazard ratios	CI95-low	CI95-high	P-value
ISHAK FIBROSIS SCORE 1,2 - Portal Fibrosis	5.147	0.280	10.014	3.82E-02
ISHAK FIBROSIS SCORE 3,4 - Fibrous Speta	12.193	2.193	22.194	1.69E-02
AJCC PATHOLOGIC TUMOR STAGEStage II	−1.977	−5.219	1.266	2.32E-01
AJCC PATHOLOGIC TUMOR STAGEStage IVA	0.774	−1.812	3.361	5.57E-01
meta.score	0.547	0.131	0.963	9.96E-03

### 3.3 Validation of the metabolic signature in proliferative status of intrahepatic cholangiocarcinoma

**Fig. 3:**
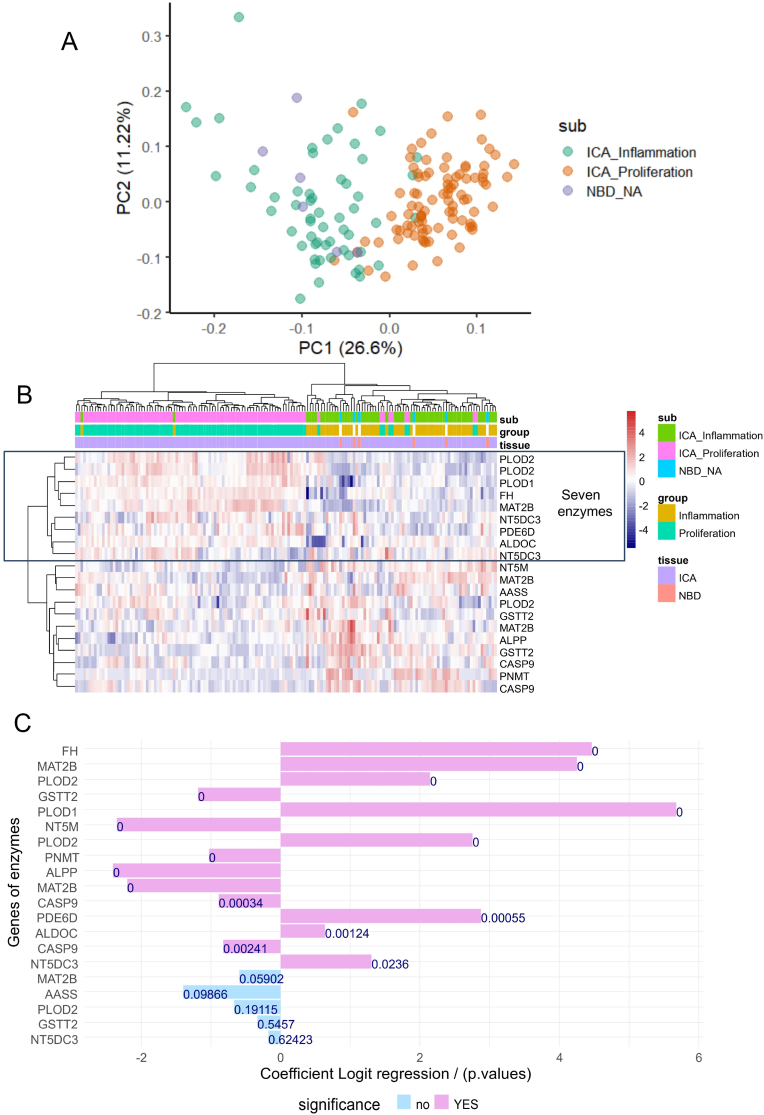
**Metabolic signature in validation cohort of ICA transcriptome:** dataset GSE32225; A/Unsupervised principal component analysis performed on expression of the 16 adverse enzymes and stratified on sample groups: NBD: normal bile duct, ICA_inflammation: intrahepatic cholangiocarcinoma of inflammation subclass, ICA_Proliferation: intrahepatic cholangiocarcinoma of proliferation subclass; B/Unsupervised clustering (Euclidean distances) and expression heatmap of the 16 adverse enzymes; C/Barplot of univariate binomial analyses according subclass proliferation status for the 16 adverse enzymes.

**Fig. 4:**
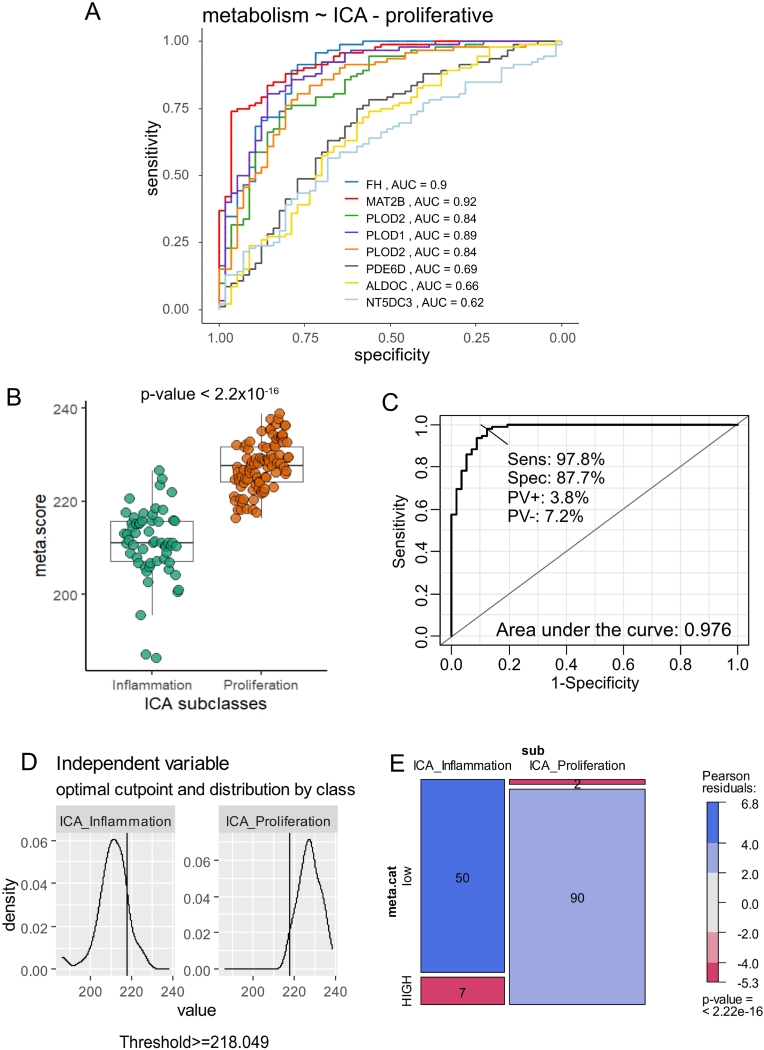
**Validation of the metabolic score in independent cohort of transcriptome and determination of the metabolism classes:** dataset GSE32225; A/Multi-ROC analyses concerning expression of up-regulated enzymes according to proliferative status of the ICA subclass; B/Computed metabolic score in ICA transcriptome and stratified according original ICA subclasses: inflammation and proliferation, p-value between groups was obtained with two sided Student t-test; C/ROC-curve performed on metabolism score according proliferative ICA subclass status (Sens: sensibility, Spe: Specificity, PV+: positive prevalent value,PV-: Negative prevalent value); D/Determination of the optimal threshold of metabolism score according proliferative/inflammation ICA subclasses; E/Mosaicplot of overlaps between original ICA sub-classication (Inflammation/Proliferation) versus metabolism score categories (low and HIGH), p-value was computed by chi.square test between the two classifications.

The expression of the sixteen enzymes with adverse effects on patient survival was investigated in the validation cohort of bulk transcriptome data (Illumina DNA-beads) for intrahepatic cholangiocarcinoma (dataset GSE32225, n = 149 ICA tumors). This transcriptome cohort identified two distinct subclasses of intrahepatic cholangiocarcinoma: proliferation (poor prognosis) and inflammation (favorable prognosis) [17]. Utilizing unsupervised principal component analysis (PCA), the expression of these sixteen adverse enzymes allowed for effective stratification of the proliferative and inflammatory subclasses of intrahepatic cholangiocarcinoma along the first principal axis, which accounted for 26.6 % of the variance (Fig. 3A). Notably, normal bile duct (NBD) samples (n = 6) were localized within the inflammatory subgroup (Fig. 3A). Unsupervised clustering based on the expression of these sixteen adverse enzymes also effectively distinguished between the two subclasses of intrahepatic cholangiocarcinoma: inflammation and proliferation (Fig. 3B). Similar to the PCA results, normal bile duct samples were classified in the inflammatory subclass (Fig. 3B). A heatmap analysis confirmed that seven enzyme were significantly overexpressed in the proliferative subclass compared to the inflammatory subclass (highlighted in blue, Fig. 3B). Univariate logit binomial analysis, with the outcome being the proliferative subclass status of intrahepatic cholangiocarcinoma, confirmed the significance of the probes for seven enzymes: FH, MAT2B, PLOD2, PLOD1, PDE6D, ALDOC, and NT5DC3 (Fig. 3C and Supplemental Table 2). Multi-ROC analysis revealed that the overexpression of MAT2B predicted the proliferative subclass of intrahepatic cholangiocarcinoma with an area under the curve (AUC) of 0.92, FH with an AUC of 0.90, PLOD1 with an AUC of 0.89, PLOD2 (two distinct quantification) with an AUC of 0.84, PDE6C with an AUC of 0.69, ALDOC with an AUC of 0.66, and NT5DC3 with an AUC of 0.62 (Fig. 4A). Based on the expression of these seven enzymes confirmed to be overexpressed in the poor prognosis of intrahepatic cholangiocarcinoma, a metabolic expression score was computed for the validation cohort GSE32225. This metabolic score was significantly different between the two subclasses of intrahepatic cholangiocarcinoma: proliferative and inflammatory (two-sided Student's t-test, p-value <2.2e-16, Fig. 4B). Optimal threshold of the metabolism score was determined to be superior or equal to 218.049 to predict proliferative class in validation cohort (Fig. 4D). Chi-square test and mosaicplot demonstrated a significant association (p-value <2.2e-16 Fig. 4E) between inflammation/proliferation classification and metabolism score categories (low and HIGH values).

### 3.4 ICA proliferative tumors harbored a general higher metabolism activation than inflammation ones by keggmetascore analysis

**Fig. 5:**
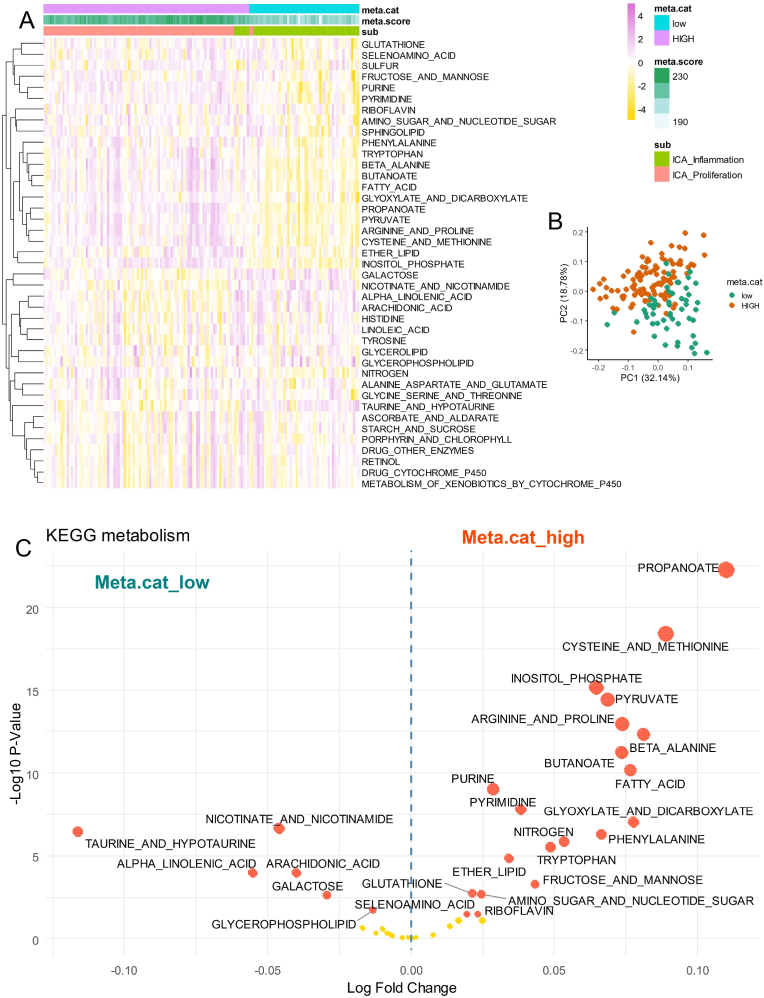
**Heterogeneity of metabolism pathway deregulations in ICA tumors:** A/heatmap of single sample GSEA (ssGSEA) analyses perfomed on 41 KEGG metabolism pathways in 149 ICA tumors from GSE32225; B/Principal component analysis performed on KEGG metabolism pathway quantification and stratify according metabolism score category classification (low and HIGH); C/Volcanoplot performed post limma analysis between metabolism categories on ssGSEA KEGG metabolism pathways of 149 ICA tumors.

In order to deeper investigated heterogeneity of metabolism activation in ICA tumors, keggmetascore analysis was developed. This process implemented in a new a R-package investigated single sample scoring of 41 metabolism pathway characterized in KEGG database. Investigation on keggmetascore analysis through ssGSEA (single sample geneset enrichment analysis) on validation cohort highlighted a general stratication (Fig. 5A) of the tumors according their inflammation/proliferation classification but also according their metabolism score categories previously determined (Fig. 4E). This stratification on metabolism score categories was confirmed by unsupervized principal component analysis based on keggmetascore results (Fig. 5B). Differential expression score analysis performed between meta.score high et low categories highligthed a general metabolism activation in tumor high as compared to low ones: especially with propanoate, cysteine and methionine, inositol phosphate, pyruvate, butanoate, fatty acid, purine metabolisms (Fig. 5C–Table 2).

### 3.5 Heterogeneity transcriptional correlated two metabolism score in ICA tumors

**Fig. 6:**
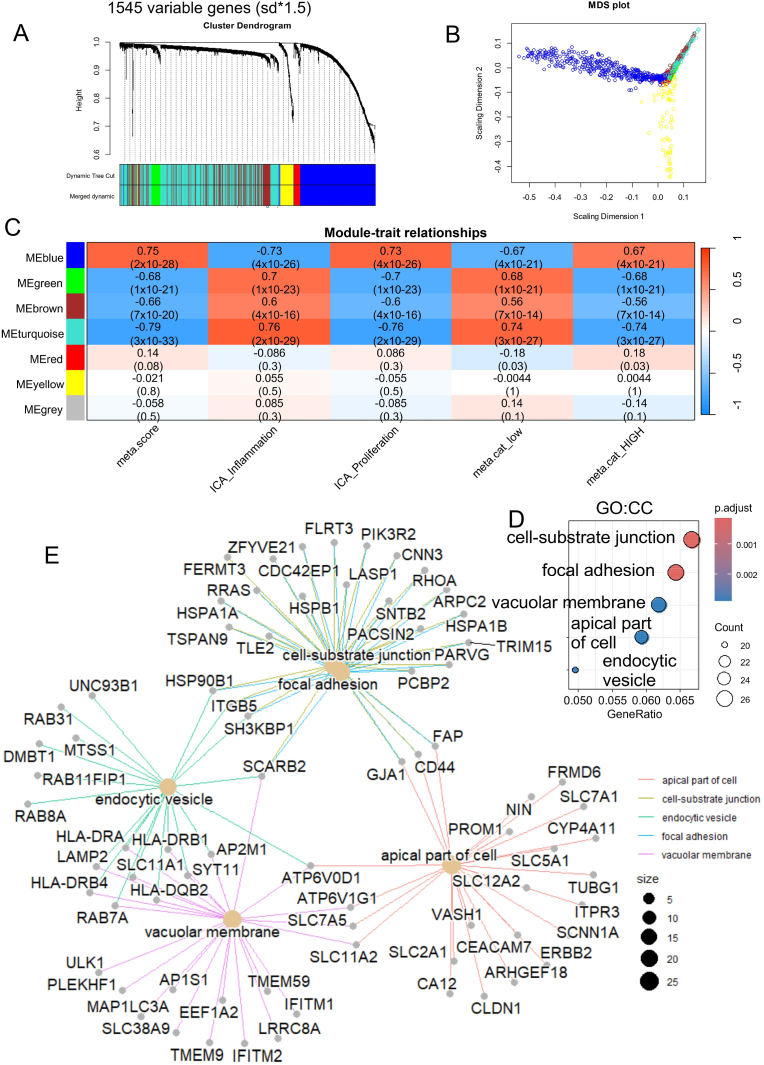
**Diversity of cellular compartments alterated in ICA tumors presenting high metabolism activity:** A/Gene regulated networks (GRN) identified in ICA tumors based on expression of the most variable genes (n = 1545 genes divided in 7 GRNs, sd: standard deviation); B/Multi-dimension scaling dimension reduction of genes classified by colored GRNs; C/Phenotype correlation traits with seven heterogenous GRNs identified in diversity of the 149 ICA tumors (Pearson coefficients and Pearson correlation test p-values were indicated in matrix of cells); D/Gene-ontology cellular component (GO::CC) functional enrichment performed on genes composing the blue GRN positively correlated to metabolism score; E/Functional enrichment score drawn on enriched cellular compartments for the blue GRN.

To investigate transcriptional heterogeniety of ICA tumors according metabolism activation, detection of gene regulatory networks (GRN, gene modules) was performed with WGCNA algorithm in tumors of validation cohort (GSE32225, n = 194). In the tumor transcriptome, the most variable genes across samples were selected based on their extreme variance: 1545 genes presented a variance over mean + 1.5 standard deviation of all genes from the microarray (Fig. 6A). This restricted variable matrix was taken as input for WGCNA analysis with a soft power optimal of 12. Seven GRN modules were identified with a major separation of the blue one versus the others (Fig. 6A–B). This blue module is composed of 464 connected genes and was found positively correlated to the metabolism quantitative score (Pearson correlation coefficient = 0.75, p-value = 2x10^−28^, Fig. 6C) and positively correlated to the ICA proliferative score (Pearson correlation coefficient = 0.73, p-value = 4x10^−26^, Fig. 6C) and positively correlated to metabolism category: HIGH (Pearson correlation coefficient = 0.67, p-value = 4x10^−21^, Fig. 6C). In opposite, green, brown, and turquoise modules were found negatively correlated to the tested phenotypes (Fig. 6C). Also, red, yellow, and grey gene modules were found less significant association with tested phenotypes (Fig. 6C). Functional enrichment was performed on blue module (positively correlated to metabolism score) with Gene Ontology Cellular Component database. This analysis highlighted implication diverse celluar compartment enriched in blue module: such as genes implicated in focal adhesion and cell-substrate junction for communication with micro-environment, some genes shared between endocytic vesicle and vacuolar membrane for intracellular trafficking, and some genes implicated in apical part of cell (Fig. 6D).

### 3.6 Among tumor microenvironment, ICA malignant cells present higher quantification of metabolic score

**Fig. 7:**
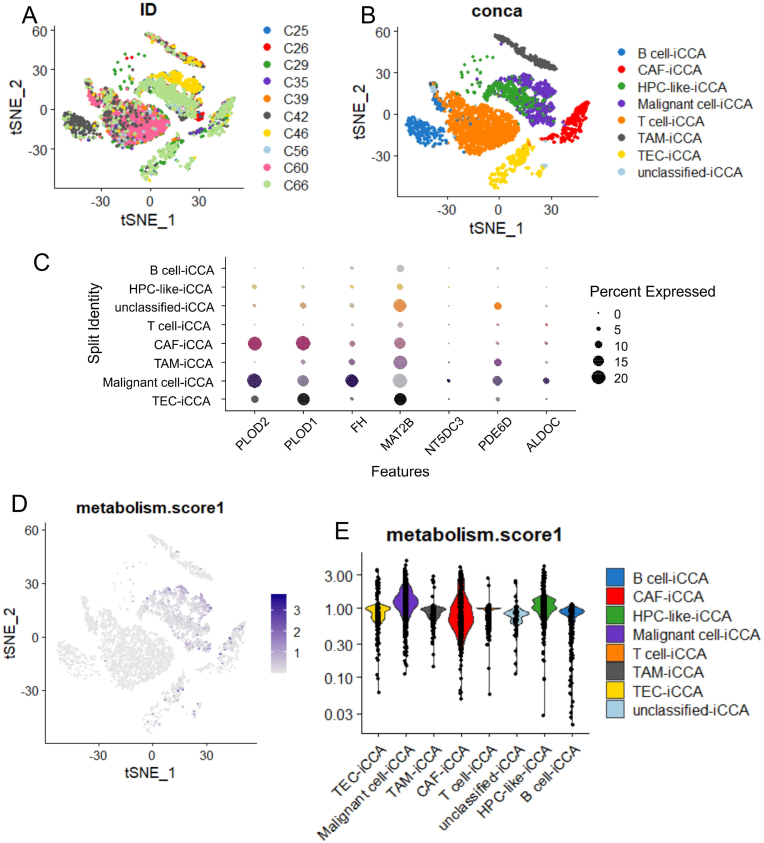
**ICA malignant cells present higher metabolic score value among tumor cell diversity:** scRNAseq GSE125449 comprising 6033 ICA cells; A/UMAP dimension reduction of scRNAseq among samples from 10 ICA patients with individual colors and annotations: C25, C26, C29, C35, C39, C42, C46, C56, C60, and C66; B/UMAP dimension reduction of scRNAseq with cell type annotation; C/Single cell expression dotplot of the validated metabolic enzymes up-regulated in adverse ICA and stratified on cell type annotation; D/UMAP dimension reduction of scRNAseq with projection of computed metabolic score; E/Violinplot of metabolic score computed in scRNAseq and stratified on ICA cell types.

Based on the training and validation cohorts of intrahepatic cholangiocarcinoma (ICA) bulk transcriptomes, seven metabolic enzymes were confirmed to be overexpressed in cases with poor prognosis: FH, MAT2B, PLOD2, PLOD1, PDE6D, ALDOC, and NT5DC3. To identify the specific cell types responsible for producing these enzymes, single-cell transcriptome experiments conducted on ten ICA tumors were analyzed (Fig. 7A, dataset GSE125449). These experiments revealed the presence of eight cell types in the tumor microenvironment of these samples: B cells, cancer-associated fibroblasts (CAFs), hepatic progenitor-like cells (HPCs), malignant cells, T cells, tumor-associated macrophages (TAMs), tumor endothelial cells (TECs), and unclassified cells (Fig. 7B). Among these cell types, the single-cell expression of the seven adverse enzymes was found to be significantly higher in the malignant cell subtype (Fig. 7C). Based on the expression of these seven enzymes, a single-cell metabolic score was computed for the ICA samples (Fig. 7D). This metabolic score was notably greater in ICA malignant cells compared to other cell types within the tumor micro-environment (Fig. 7D–E, Table 4).

**Table 4:** **Comparison of single cell metabolic score across intrahepatic cholangiocarcinoma cell types:** means of single cell metabolic score was computed in each cell types detected in ICA tumors, two sided Student t-tests were performed between Malignant cells and others cell types.

Comparison	mean Malignant cells	means other cell types	p_value
Malignant cell-iCCA vsTEC-iCCA	0.297	−0.065	7.74E-29
Malignant cell-iCCA vs TAM-iCCA	0.297	−0.133	3.22E-92
Malignant cell-iCCA vs CAF-iCCA	0.297	−0.065	6.58E-24
Malignant cell-iCCA vs T cell-iCCA	0.297	−0.074	1.34E-96
Malignant cell-iCCA vs unclassified-iCCA	0.297	−0.243	1.34E-29
Malignant cell-iCCA vs HPC-like-iCCA	0.297	0.111	3.91E-18
Malignant cell-iCCA vs B cell-iCCA	0.297	−0.305	8.37E-119

### 3.7 Gene-drug interactions for targeting the seven activated enzymes from metabolism scor of ICA tumors

To identify potential enzyme targets of drugs in the seven activated enzymes, Dgldb version durg-gene interaction database [43] was screened by regular expression on the gene names of the seven activated enzymes: FH, MAT2B, PLOD2, PLOD1, PDE6D, ALDOC, and NT5DC3. For the seven enzymes, eight drug interactions were detected for four enzymes (Table 5). FH was found to interact with four unapproved drugs and three of them were classed as inhibitors: SBI-0206965 with greater interaction score (AMPK-ULK1 inhibitor) [44], SBP-7455 with intermediary one (ULK1/2 Autophagy Inhibitor) [45], and COMPOUND R-16 with smaller one [46]. PDE6D was found to interact with two inhibitor aproved drugs presenting both small interaction scores: aspirin and pentoxifylline. Finally, PLOD1 was found to interact with ascorbic acid but its type of interaction is not determined and ALDOC was found to strongly interact with an antibody named Vantictumab (Table 5) designed as anti-frizzled (FZD) [47].

**Table 5:** **Table of gene-drug interaction found in DGldb5.0 database for the seven activated enzymes in ICA tumors (G2P:** GuideToPharmacology) (#): drugs already approved.

Gene name	Database	Interaction type	Interaction score	Drug name
ALDOC	G2P	antibody	10,502	VANTICTUMAB
FH	G2P	inhibitor	13,127	SBI-0206965
FH	NCI	NULL	3751	DIETHYLDITHIOCARBAMATE
FH	G2P	inhibitor	2625	COMPOUND R-16 [PMID: 21967808]
FH	G2P	inhibitor	6563	SBP-7455
PDE6D	ChEMBL	inhibitor	0,186	ASPIRIN (#)
PDE6D	ChEMBL	inhibitor	0,750	PENTOXIFYLLINE (#)
PLOD1	TTD	NULL	2020	ASCORBIC ACID (#)

## 4 Discussion

We established in intrahepatic cholangiocarcinoma tumors, a metabolic score composed of the expression of seven metabolic enzymes which predict the early adverse prognosis of the disease.

The transcriptional analysis of intrahepatic cholangiocarcinoma (ICA) revealed significant alterations in seven metabolic enzymes that play crucial roles in tumor biology and therapeutic strategies. To deeper investigate metabolism, keggmetascore R-package was developed to perform single sample scoring in transcriptomic and proteomic experiments. Application of keggmetascore scoring to ICA transcriptome cohort validated a general activated of metabolism pathways in proliferative ICA tumors of bad prognosis and also validated metabolism score classification based on expression of the seven activated enzymes in ICA tumors from bad prognosis. This application validated major activation of propanoate, cysteine and methionine, inositol phosphate, pyruvate, butanoate, fatty acid, purine metabolisms in ICA tumors of bad prognosis which in accordance with known general metabolism reprogramming of tumor cells during cholangiocarcinoma [7]. Metabolism score ICA tumors was found connected to a gene regulatory network implicating many cell sub-compartments such as cell substrate junction and focal adhesion for communication with micro-environment that could be associated to the progression of the disease [48], endocytic vesicle and vacuolar membrane for intracellular trafficking potentially associated to increase uptake of divers metabolites by the tumoral cells [7], and apical part of cell with potential deregulation epthelial cell polarization [49].

Among these seven enzymes activated during bad prognosis of ICA, methionine adenosyltransferase 2B (MAT2B) is particularly noteworthy as it is essential for producing S-adenosylmethionine, a key methyl donor in various biological processes. MAT2B encodes the regulatory subunit MATβ, which modulates MATα2 activity. A shift from MAT1A to MAT2A/MAT2B is observed in multiple liver diseases, indicating its involvement in liver growth and dedifferentiation [50].

Another critical enzyme is fumarate hydratase (FH), which functions in the tricarboxylic acid cycle and is localized in the mitochondrial matrix. The cytosolic form of FH is implicated in the DNA damage response, especially in dealing with double-strand breaks, and is also involved in histone demethylation. In the context of liver pathophysiology, anti-FH autoantibodies serve as prognostic biomarkers for Acute-on-Chronic Liver Failure [51]. Genetic defects in FH can lead to elevated fumarate levels, which stabilize HIF-1α and activate pathways that promote angiogenesis, further linking it to tumor progression [52].

The identification of fructose-bisphosphate aldolase C (ALDOC) as part of the adverse metabolic transcriptional program in ICA is significant due to its crucial role in the glycolytic pathway. ALDOC catalyzes the conversion of fructose-1,6-bisphosphate to glyceraldehyde-3-phosphate and dihydroxyacetone phosphate, indicating a metabolic shift toward glycolysis [53,54]. This upregulation suggests a preference for glycolytic energy production, commonly associated with the Warburg effect, which supports tumor growth and survival by providing essential metabolites for rapid cell proliferation. The overexpression of ALDOC may also serve as a biomarker for poor prognosis in ICA patients [53,54].

Similarly, procollagen-lysine, 2-oxoglutarate 5-dioxygenase 2 (PLOD2) plays a pivotal role in collagen post-translational modification. By hydroxylating lysine residues, PLOD2 contributes to collagen stability and cross-linking, which are essential for maintaining the structural integrity of the extracellular matrix (ECM). In cancer disease, increased PLOD2 expression indicates enhanced collagen deposition, fostering a desmoplastic reaction that promotes tumor progression by creating a supportive microenvironment for cancer cell proliferation and invasion [55]. Elevated PLOD2 levels have been linked to poor prognosis, making it a potential biomarker and therapeutic target in ICA.

The role of procollagen-lysine, 2-oxoglutarate 5-dioxygenase 1 (PLOD1) is similarly important. Like PLOD2, PLOD1 is involved in collagen hydroxylation, and its upregulation in ICA suggests increased collagen synthesis, leading to a dense ECM, as demonstrated in other cancer types [56]. This desmoplastic response may facilitate tumor progression and immune evasion, as the altered ECM can create barriers against immune cell infiltration [55]. Elevated PLOD1 expression is associated with poor prognosis, indicating its potential utility as a biomarker. Targeting PLOD1 may disrupt collagen synthesis and enhance treatment efficacy by re-sensitizing tumors to conventional therapies.

Phosphodiesterase 6D (PDE6D) also stands out due to its role in hydrolyzing cyclic nucleotides, which are crucial for signaling pathways involved in cell proliferation, differentiation, oxidative stress and apoptosis. Increased PDE6D levels in ICA may enhance tumor growth by modulating intracellular cyclic nucleotide levels, influencing pathways such as MAPK/ERK and PI3K/Akt, which are often dysregulated in cancer [[57], [58], [59], [60], [61], [62]]. This dysregulation may also facilitate tumor progression by altering interactions between cancer cells and the surrounding stroma, contributing to an immunosuppressive environment. Targeting PDE6D presents a promising therapeutic strategy, as its inhibition could restore normal cyclic nucleotide levels and enhance the efficacy of existing treatments.

Lastly, the identification of 5′-nucleotidase domain containing 3 (NT5DC3) in the adverse metabolic transcriptional program of ICA is significant for several reasons. NT5DC3 has been shown to suppress cell proliferation and induce cell cycle arrest [63], as well as being an inhibitor of tumour suppressors such as p53 [64], making it an interesting biomarker in the study of ICA, since targeting NT5DC3 could reduce adenosine levels, restore immune function, and enhance the effectiveness of immunotherapies.

In summary, our study identifies a network of metabolic enzymes—MAT2B, FH, ALDOC, PLOD1, PLOD2, PDE6D, and NT5DC3—that play crucial roles in the metabolic reprogramming of ICA. These enzymes not only contribute to tumor biology but also present potential biomarkers and therapeutic targets. Notably, several of these enzymes are druggable: for example, PLOD1 and PLOD2, which are involved in collagen crosslinking, can be targeted by specific inhibitors such as minoxidil, which disrupts collagen maturation and tumor-stroma interactions [65]. MAT2B, a key regulator of methionine metabolism, can be inhibited by cycloleucine or FIDAS-5 to interfere with methylation processes in cancer cells [66,67]. In gene-drug interaction database, fumarate hydratase (FH) was found to interact with SBI-0206965 (AMPK-ULK1 inhibitor) [44], with SBP-7455 (ULK1/2 Autophagy Inhibitor) [45], and COMPOUND R-16 [46]. ALDOC was found to strongly interact with an antibody named Vantictumab (Table 5) designed as anti-frizzled (FZD) [47].

Targeting these enzymes within a druggable metabolic network provides a promising strategy for therapeutic intervention (Supplementary Fig. 2). Further research is needed to explore the specific mechanisms and interactions within this metabolic framework, paving the way for targeted treatments that can disrupt the adverse metabolic programs sustaining tumor growth, and ultimately improve patient outcomes.

## CRediT authorship contribution statement

**Christophe Desterke:** Writing – original draft, Software, Methodology, Formal analysis, Data curation. **Raquel Francés:** Validation, Investigation, Formal analysis. **Claudia Monge:** Visualization, Validation. **Yuanji Fu:** Validation, Software. **Agnès Marchio:** Visualization, Validation, Software. **Pascal Pineau:** Writing – review & editing, Visualization, Supervision. **Jorge Mata-Garrido:** Writing – review & editing, Writing – original draft, Resources, Project administration, Investigation, Funding acquisition, Conceptualization.

## Data availability

- Scripts in R programming language employed during this publication are available at the following web address: https://github.com/cdesterke/ica_metabolism (accessed on 2024, September 30th).

-”keggmetascore” developed application is available for R installation at the following web address: https://github.com/cdesterke/keggmetascore (accessed on 2025, January 20th).

## Declaration of competing interest

The authors declare that there are no conflicts of interest regarding the publication of this manuscript. No funding, grants, or other financial support were received that could have influenced the research presented in this paper. Additionally, the authors have no affiliations, financial involvement, or personal relationships with any organization or entity that could be perceived as influencing the content of this submission to *Biochemistry and Biophysics Reports*.

## References

[1] Gans J.H., Lipman J., Golowa Y., Kinkhabwala M., Kaubisch A. (2019). Hepatic cancers overview: surgical and chemotherapeutic options, how do Y-90 microspheres fit in?. Semin. Nucl. Med..

[2] Massarweh N.N., El-Serag H.B. (2017). Epidemiology of hepatocellular carcinoma and intrahepatic cholangiocarcinoma. Cancer Control.

[3] Forner A., Reig M., Bruix J. (2018). Hepatocellular carcinoma. Lancet.

[4] Sarcognato S., Sacchi D., Fassan M., Fabris L., Cadamuro M., Zanus G., Cataldo I., Capelli P., Baciorri F., Cacciatore M., Guido M. (2021). Cholangiocarcinoma. Pathologica.

[5] El-Diwany R., Pawlik T.M., Ejaz A. (2019). Intrahepatic cholangiocarcinoma. Surg. Oncol. Clin..

[6] Mranda G.M., Xiang Z.-P., Liu J.-J., Wei T., Ding Y. (2022). Advances in prognostic and therapeutic targets for hepatocellular carcinoma and intrahepatic cholangiocarcinoma: the hippo signaling pathway. Front. Oncol..

[7] Raggi C., Taddei M.L., Rae C., Braconi C., Marra F. (2022). Metabolic reprogramming in cholangiocarcinoma. J. Hepatol..

[8] Yang C., Huang X., Liu Z., Qin W., Wang C. (2020). Metabolism-associated molecular classification of hepatocellular carcinoma. Mol. Oncol..

[9] Pope E.D., Kimbrough E.O., Vemireddy L.P., Surapaneni P.K., Copland J.A., Mody K. (2019). Aberrant lipid metabolism as a therapeutic target in liver cancer. Expert Opin. Ther. Targets.

[10] Xu K., Xia P., Chen X., Ma W., Yuan Y. (2023). ncRNA-mediated fatty acid metabolism reprogramming in HCC. Trends Endocrinol. Metabol..

[11] Kipp B.R., Voss J.S., Kerr S.E., Barr Fritcher E.G., Graham R.P., Zhang L., Highsmith W.E., Zhang J., Roberts L.R., Gores G.J., Halling K.C. (2012). Isocitrate dehydrogenase 1 and 2 mutations in cholangiocarcinoma. Hum. Pathol..

[12] Delahousse J., Verlingue L., Broutin S., Legoupil C., Touat M., Doucet L., Ammari S., Lacroix L., Ducreux M., Scoazec J.-Y., Malka D., Paci A., Hollebecque A. (2018). Circulating oncometabolite D-2-hydroxyglutarate enantiomer is a surrogate marker of isocitrate dehydrogenase–mutated intrahepatic cholangiocarcinomas. Eur. J. Cancer.

[13] Snaebjornsson M.T., Janaki-Raman S., Schulze A. (2020). Greasing the wheels of the cancer machine: the role of lipid metabolism in cancer. Cell Metab..

[14] Huang T., Cao H., Liu C., Sun X., Dai S., Liu L., Wang Y., Guo C., Wang X., Gao Y., Tang W., Xia Y. (2024). MAL2 reprograms lipid metabolism in intrahepatic cholangiocarcinoma via EGFR/SREBP-1 pathway based on single-cell RNA sequencing. Cell Death Dis..

[15] Liu J., Lichtenberg T., Hoadley K.A., Poisson L.M., Lazar A.J., Cherniack A.D., Kovatich A.J., Benz C.C., Levine D.A., Lee A.V., Omberg L., Wolf D.M., Shriver C.D., Thorsson V., Hu H., Cancer Genome Atlas Research Network (2018). An integrated TCGA pan-cancer clinical data resource to drive high-quality survival outcome analytics. Cell.

[16] Li B., Dewey C.N. (2011). RSEM: accurate transcript quantification from RNA-Seq data with or without a reference genome. BMC Bioinf..

[17] Sia D., Hoshida Y., Villanueva A., Roayaie S., Ferrer J., Tabak B., Peix J., Sole M., Tovar V., Alsinet C., Cornella H., Klotzle B., Fan J.-B., Cotsoglou C., Thung S.N., Fuster J., Waxman S., Garcia-Valdecasas J.C., Bruix J., Schwartz M.E., Beroukhim R., Mazzaferro V., Llovet J.M. (2013). Integrative molecular analysis of intrahepatic cholangiocarcinoma reveals 2 classes that have different outcomes. Gastroenterology.

[18] Davis S., Meltzer P.S. (2007). GEOquery: a bridge between the gene expression Omnibus (GEO) and BioConductor. Bioinformatics.

[19] Ma L., Hernandez M.O., Zhao Y., Mehta M., Tran B., Kelly M., Rae Z., Hernandez J.M., Davis J.L., Martin S.P., Kleiner D.E., Hewitt S.M., Ylaya K., Wood B.J., Greten T.F., Wang X.W. (2019). Tumor cell biodiversity drives microenvironmental reprogramming in liver cancer. Cancer Cell.

[20] Barrett T., Wilhite S.E., Ledoux P., Evangelista C., Kim I.F., Tomashevsky M., Marshall K.A., Phillippy K.H., Sherman P.M., Holko M., Yefanov A., Lee H., Zhang N., Robertson C.L., Serova N., Davis S., Soboleva A. (2013). NCBI GEO: archive for functional genomics data sets--update. Nucleic Acids Res..

[21] Butler A., Hoffman P., Smibert P., Papalexi E., Satija R. (2018). Integrating single-cell transcriptomic data across different conditions, technologies, and species. Nat. Biotechnol..

[22] Corcoran C.C., Grady C.R., Pisitkun T., Parulekar J., Knepper M.A. (2017). From 20th century metabolic wall charts to 21st century systems biology: database of mammalian metabolic enzymes. Am. J. Physiol. Ren. Physiol..

[23] Cunningham F., Allen J.E., Allen J., Alvarez-Jarreta J., Amode M.R., Armean I.M., Austine-Orimoloye O., Azov A.G., Barnes I., Bennett R., Berry A., Bhai J., Bignell A., Billis K., Boddu S., Brooks L., Charkhchi M., Cummins C., Da Rin Fioretto L., Davidson C., Dodiya K., Donaldson S., El Houdaigui B., El Naboulsi T., Fatima R., Giron C.G., Genez T., Martinez J.G., Guijarro-Clarke C., Gymer A., Hardy M., Hollis Z., Hourlier T., Hunt T., Juettemann T., Kaikala V., Kay M., Lavidas I., Le T., Lemos D., Marugán J.C., Mohanan S., Mushtaq A., Naven M., Ogeh D.N., Parker A., Parton A., Perry M., Piližota I., Prosovetskaia I., Sakthivel M.P., Salam A.I.A., Schmitt B.M., Schuilenburg H., Sheppard D., Pérez-Silva J.G., Stark W., Steed E., Sutinen K., Sukumaran R., Sumathipala D., Suner M.-M., Szpak M., Thormann A., Tricomi F.F., Urbina-Gómez D., Veidenberg A., Walsh T.A., Walts B., Willhoft N., Winterbottom A., Wass E., Chakiachvili M., Flint B., Frankish A., Giorgetti S., Haggerty L., Hunt S.E., Iisley G.R., Loveland J.E., Martin F.J., Moore B., Mudge J.M., Muffato M., Perry E., Ruffier M., Tate J., Thybert D., Trevanion S.J., Dyer S., Harrison P.W., Howe K.L., Yates A.D., Zerbino D.R., Flicek P. (2022). Ensembl 2022. Nucleic Acids Res..

[24] Ritchie M.E., Phipson B., Wu D., Hu Y., Law C.W., Shi W., Smyth G.K. (2015). Limma powers differential expression analyses for RNA-sequencing and microarray studies. Nucleic Acids Res..

[25] Desterke C., Xiang Y., Elhage R., Duruel C., Chang Y., Hamaï A. (2023). Ferroptosis inducers upregulate PD-L1 in recurrent triple-negative breast cancer. Cancers.

[26] Kassambara A., Kosinski M., Biecek P., Fabian S. (2021). survminer: drawing Survival Curves using “ggplot2,”. https://cran.r-project.org/web/packages/survminer/index.html.

[27] Ishak K., Baptista A., Bianchi L., Callea F., De Groote J., Gudat F., Denk H., Desmet V., Korb G., MacSween R.N.M., Phillips M.J., Portmann B.G., Poulsen H., Scheuer P.J., Schmid M., Thaler H. (1995). Histological grading and staging of chronic hepatitis. J. Hepatol..

[28] Therneau T.M., Grambsch P.M. (2000). http://www.springer.com/us/book/9780387987842.

[29] Harrell F.E., Harrell Frank E. (2015). Regression Modeling Strategies: with Applications to Linear Models, Logistic and Ordinal Regression, and Survival Analysis.

[30] Desterke C., Cosialls E., Xiang Y., Elhage R., Duruel C., Chang Y., Hamaï A. (2023). Adverse crosstalk between extracellular matrix remodeling and ferroptosis in basal breast cancer. Cells.

[31] Thiele C., Hirschfeld G. (2021). Cutpointr : improved estimation and validation of optimal cutpoints in *R*. J. Stat. Software.

[32] Zeileis A., Meyer D., Hornik K. (2007). Residual-based shadings for visualizing (conditional) independence. J. Comput. Graph Stat..

[33] Liberzon A., Birger C., Thorvaldsdóttir H., Ghandi M., Mesirov J.P., Tamayo P. (2015). The Molecular Signatures Database (MSigDB) hallmark gene set collection. Cell Syst.

[34] Ogata H., Goto S., Sato K., Fujibuchi W., Bono H., Kanehisa M. (1999). KEGG: Kyoto Encyclopedia of genes and Genomes. Nucleic Acids Res..

[35] (2019). The UniProt Consortium, UniProt: a worldwide hub of protein knowledge. Nucleic Acids Res..

[36] Hänzelmann S., Castelo R., Guinney J. (2013). GSVA: gene set variation analysis for microarray and RNA-seq data. BMC Bioinf..

[37] Horvath S. (2011).

[38] Langfelder P., Horvath S. (2008). WGCNA: an R package for weighted correlation network analysis. BMC Bioinf..

[39] Wu T., Hu E., Xu S., Chen M., Guo P., Dai Z., Feng T., Zhou L., Tang W., Zhan L., Fu X., Liu S., Bo X., Yu G. (2021). clusterProfiler 4.0: a universal enrichment tool for interpreting omics data. Innovation.

[40] Xu S., Hu E., Cai Y., Xie Z., Luo X., Zhan L., Tang W., Wang Q., Liu B., Wang R., Xie W., Wu T., Xie L., Yu G. (2024). Using clusterProfiler to characterize multiomics data. Nat. Protoc..

[41] Ashburner M., Ball C.A., Blake J.A., Botstein D., Butler H., Cherry J.M., Davis A.P., Dolinski K., Dwight S.S., Eppig J.T., Harris M.A., Hill D.P., Issel-Tarver L., Kasarskis A., Lewis S., Matese J.C., Richardson J.E., Ringwald M., Rubin G.M., Sherlock G. (2000). Gene ontology: tool for the unification of biology. The Gene Ontology Consortium. Nat. Genet..

[42] Kobak D., Berens P. (2019). The art of using t-SNE for single-cell transcriptomics. Nat. Commun..

[43] Cannon M., Stevenson J., Stahl K., Basu R., Coffman A., Kiwala S., McMichael J.F., Kuzma K., Morrissey D., Cotto K., Mardis E.R., Griffith O.L., Griffith M., Wagner A.H. (2024). DGIdb 5.0: rebuilding the drug-gene interaction database for precision medicine and drug discovery platforms. Nucleic Acids Res..

[44] Yu L., Shi Q., Jin Y., Liu Z., Li J., Sun W. (2021). Blockage of AMPK-ULK1 pathway mediated autophagy promotes cell apoptosis to increase doxorubicin sensitivity in breast cancer (BC) cells: an in vitro study. BMC Cancer.

[45] Ren H., Bakas N.A., Vamos M., Chaikuad A., Limpert A.S., Wimer C.D., Brun S.N., Lambert L.J., Tautz L., Celeridad M., Sheffler D.J., Knapp S., Shaw R.J., Cosford N.D.P. (2020). Design, synthesis, and characterization of an orally active dual-specific ULK1/2 autophagy inhibitor that synergizes with the PARP inhibitor olaparib for the treatment of triple-negative breast cancer. J. Med. Chem..

[46] Milkiewicz K.L., Aimone L.D., Albom M.S., Angeles T.S., Chang H., Grobelny J.V., Husten J., Losardo C., Miknyoczki S., Murthy S., Rolon-Steele D., Underiner T.L., Weinberg L.R., Worrell C.S., Zeigler K.S., Dorsey B.D. (2011). Improvement in oral bioavailability of 2,4-diaminopyrimidine c-Met inhibitors by incorporation of a 3-amidobenzazepin-2-one group. Bioorg. Med. Chem..

[47] Diamond J.R., Becerra C., Richards D., Mita A., Osborne C., O'Shaughnessy J., Zhang C., Henner R., Kapoun A.M., Xu L., Stagg B., Uttamsingh S., Brachmann R.K., Farooki A., Mita M. (2020). Phase Ib clinical trial of the anti-frizzled antibody vantictumab (OMP-18R5) plus paclitaxel in patients with locally advanced or metastatic HER2-negative breast cancer. Breast Cancer Res. Treat..

[48] Song X., Xu H., Wang P., Wang J., Affo S., Wang H., Xu M., Liang B., Che L., Qiu W., Schwabe R.F., Chang T.T., Vogl M., Pes G.M., Ribback S., Evert M., Chen X., Calvisi D.F. (2021). Focal adhesion kinase (FAK) promotes cholangiocarcinoma development and progression via YAP activation. J. Hepatol..

[49] Bou Malham V., Benzoubir N., Vaquero J., Desterke C., Agnetti J., Song P.X., Gonzalez‐Sanchez E., Arbelaiz A., Jacques S., Di Valentin E., Rahmouni S., Tan T.Z., Samuel D., Thiery J.P., Sebagh M., Fouassier L., Gassama‐Diagne A. (2023). Intrinsic cancer cell phosphoinositide 3‐kinase δ regulates fibrosis and vascular development in cholangiocarcinoma. Liver Int..

[50] Murray B., Barbier-Torres L., Fan W., Mato J.M., Lu S.C. (2019). Methionine adenosyltransferases in liver cancer. World J. Gastroenterol..

[51] Wei L., Wang T., Chen S., Liu Y., Huang X., Zheng S., Xu B., Ren F., Liu M. (2023). Serum anti-fumarate hydratase autoantibody as a biomarker for predicting prognosis of acute-on-chronic liver failure. Gut Liver.

[52] Luo H., Wang Q., Yang F., Liu R., Gao Q., Cheng B., Lin X., Huang L., Chen C., Xiang J., Wang K., Qin B., Tang N. (2023). Signaling metabolite succinylacetone activates HIF-1α and promotes angiogenesis in GSTZ1-deficient hepatocellular carcinoma. JCI Insight.

[53] Maruyama R., Nagaoka Y., Ishikawa A., Akabane S., Fujiki Y., Taniyama D., Sentani K., Oue N. (2022). Overexpression of aldolase, fructose-bisphosphate C and its association with spheroid formation in colorectal cancer. Pathol. Int..

[54] Li Y.-J., Huang T.-H., Hsiao M., Lin B.-R., Cheng S.-J., Yang C.-N., Lai W.-T., Wu T.-S., Fan J.-R., Kuo M.Y.-P., Chang C.-C. (2016). Suppression of fructose-bisphosphate aldolase C expression as a predictor of advanced oral squamous cell carcinoma. Head Neck.

[55] Gong S., Wu C., Köhler F., Meixensberger J., Schopow N., Kallendrusch S. (2022). Procollagen-lysine, 2-oxoglutarate 5-dioxygenase family: novel prognostic biomarkers and tumor microenvironment regulators for lower-grade glioma. Front. Cell. Neurosci..

[56] Li B., Yang H., Shen B., Huang J., Qin Z. (2021). Procollagen-lysine, 2-oxoglutarate 5-dioxygenase 1 increases cellular proliferation and colony formation capacity in lung cancer via activation of E2F transcription factor 1. Oncol. Lett..

[57] Jenal U., Reinders A., Lori C. (2017). Cyclic di-GMP: second messenger extraordinaire. Nat. Rev. Microbiol..

[58] Li T., Huang T., Du M., Chen X., Du F., Ren J., Chen Z.J. (2021). Phosphorylation and chromatin tethering prevent cGAS activation during mitosis. Science.

[59] Zhao Q., Wei Y., Pandol S.J., Li L., Habtezion A. (2018). STING signaling promotes inflammation in experimental Acute pancreatitis. Gastroenterology.

[60] Prasad H., Shenoy A.R., Visweswariah S.S. (2020). Cyclic nucleotides, gut physiology and inflammation. FEBS J..

[61] Li J., Yang S., Billiar T.R. (2000). Cyclic nucleotides suppress tumor necrosis factor alpha-mediated apoptosis by inhibiting caspase activation and cytochrome c release in primary hepatocytes via a mechanism independent of Akt activation. J. Biol. Chem..

[62] Curatola A.M., Xu J., Hendricks-Munoz K.D. (2011). Cyclic GMP protects endothelial progenitors from oxidative stress. Angiogenesis.

[63] Cui Y., Wen Y., Lv C., Zhao D., Yang Y., Qiu H., Wang C. (2022). Decreased RNA-binding protein IGF2BP2 downregulates NT5DC2, which suppresses cell proliferation, and induces cell cycle arrest and apoptosis in diffuse large B-cell lymphoma cells by regulating the p53 signaling pathway. Mol. Med. Rep..

[64] Jin X., Liu X., Zhang Z., Xu L. (2020). NT5DC2 suppression restrains progression towards metastasis of non-small-cell lung cancer through regulation p53 signaling. Biochem. Biophys. Res. Commun..

[65] Knitlova J., Doubkova M., Plencner M., Vondrasek D., Eckhardt A., Ostadal M., Musilkova J., Bacakova L., Novotny T. (2021). Minoxidil decreases collagen I deposition and tissue-like contraction in clubfoot-derived cells: a way to improve conservative treatment of relapsed clubfoot?. Connect. Tissue Res..

[66] Sun H., Kang J., Su J., Zhang J., Zhang L., Liu X., Zhang J., Wang F., Lu Z., Xing X., Chen H., Zhang Y. (2019). Methionine adenosyltransferase 2A regulates mouse zygotic genome activation and morula to blastocyst transition. Biol. Reprod..

[67] Zhang W., Sviripa V., Chen X., Shi J., Yu T., Hamza A., Ward N.D., Kril L.M., Vander Kooi C.W., Zhan C.-G., Evers B.M., Watt D.S., Liu C., Fluorinated N. (2013). N-dialkylaminostilbenes repress colon cancer by targeting methionine S-adenosyltransferase 2A. ACS Chem. Biol..

