# Supplementary material for: Single-cell RNAseq reveals adverse metabolic transcriptional program in intrahepatic cholangiocarcinoma malignant cells
Source: Biochem Biophys Rep. 2025 Feb 15;41:101949. doi: 10.1016/j.bbrep.2025.101949 (PMC11872667; doi:10.1016/j.bbrep.2025.101949)
Supplement: Multimedia component 1 [file mmc1.docx]

**Supplemental material**

**Supplementary Figure 1: Evaluation of multivariate overall survival model in transcriptome of TCGA-ICA samples:** A/Global and individual Shoenfeld test performed on cox resuduals of the covariates integrated in the multivariate overall survival model; B/ Prediction of metabolic score adjusted on Ishak fibrosis score and on tumor staging in the multivariate overall survival model.


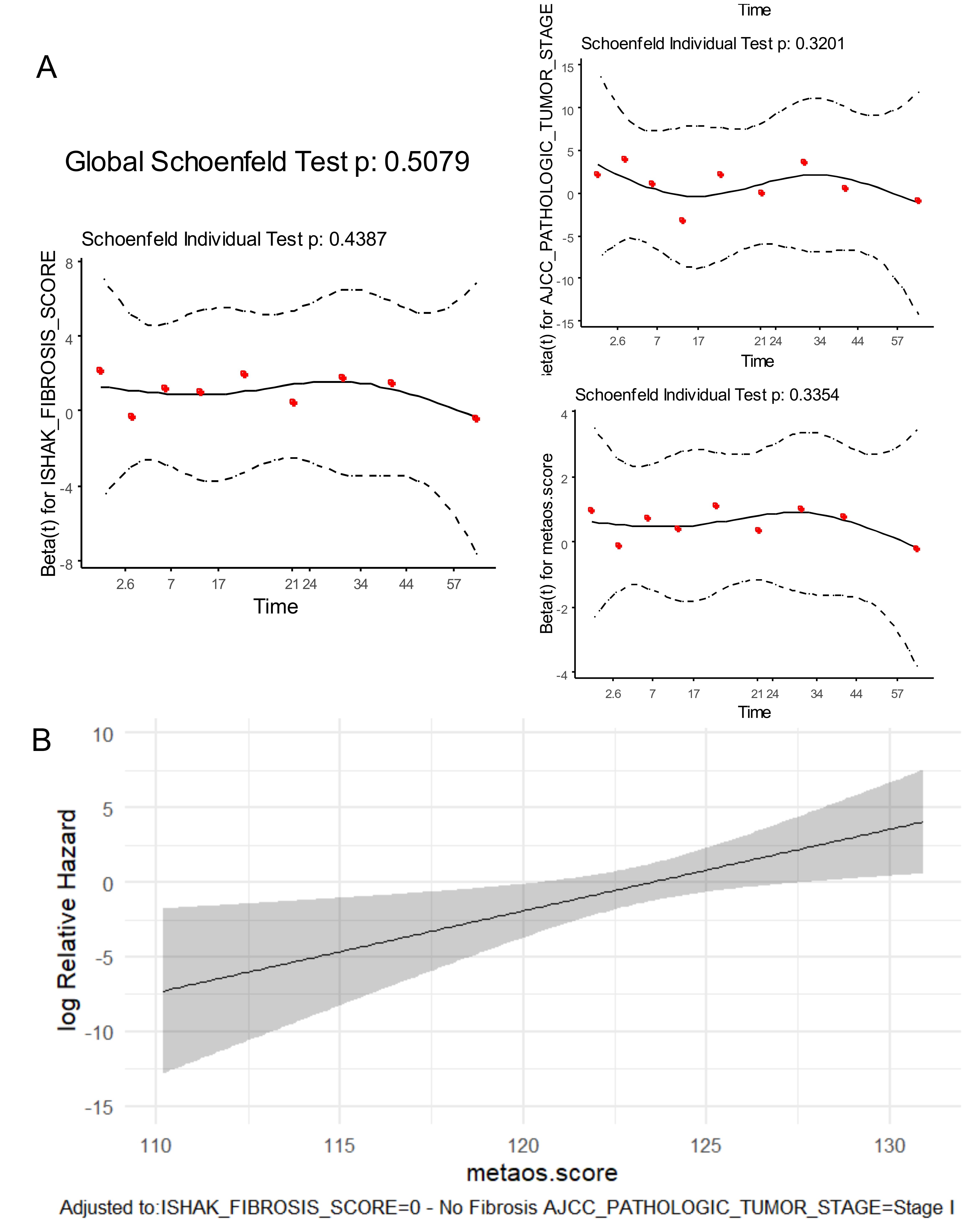


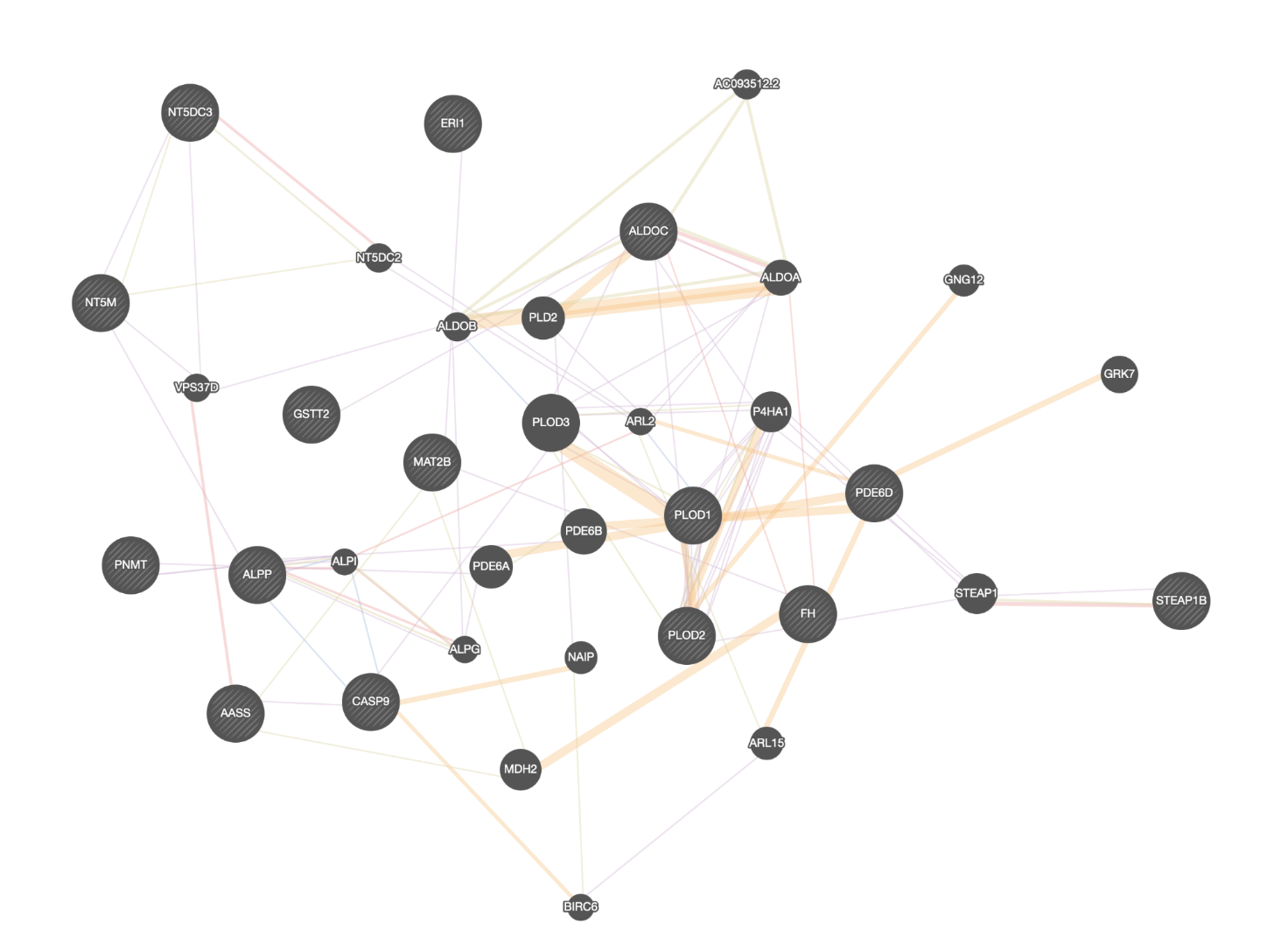
**Supplementary Figure 2.** GO representation of the network of druggable enzymes for possible future ICA treatments.

**Supplemental Table 1: Univariate Cox overall survival analyses for the 16 significant and adverse enzyme for their expression in training cohort TCGA-ICA**

| **identifiers** | **Hazard ratios** | **P-values** | **significance** | **prognosis** |
| --- | --- | --- | --- | --- |
| ACSM4 | 13.505 | 0.014 | YES | unfavorable |
| ALDOC | 2.060 | 0.014 | YES | unfavorable |
| CASP9 | 5.520 | 0.014 | YES | unfavorable |
| PLOD2 | 1.764 | 0.018 | YES | unfavorable |
| STEAP1B | 1.512 | 0.023 | YES | unfavorable |
| PDE6D | 8.856 | 0.023 | YES | unfavorable |
| ERI1 | 9.871 | 0.025 | YES | unfavorable |
| ALPP | 2.078 | 0.027 | YES | unfavorable |
| PNMT | 1.913 | 0.031 | YES | unfavorable |
| NT5M | 1.775 | 0.034 | YES | unfavorable |
| NT5DC3 | 2.752 | 0.035 | YES | unfavorable |
| MAT2B | 5.962 | 0.037 | YES | unfavorable |
| GSTT2 | 1.444 | 0.038 | YES | unfavorable |
| PLOD1 | 2.494 | 0.041 | YES | unfavorable |
| FH | 2.344 | 0.041 | YES | unfavorable |
| AASS | 1.663 | 0.044 | YES | unfavorable |

**Supplemental Table 2**

| **predictors** | **odds.ratios** | **p.values** | **significance** | **risk** |
| --- | --- | --- | --- | --- |
| ILMN_1719392_FH | 87.27 | 1.51E-09 | YES | POSITIVE |
| ILMN_1811367_MAT2B | 70.34 | 1.87E-09 | YES | POSITIVE |
| ILMN_2410924_PLOD2 | 8.57 | 3.41E-09 | YES | POSITIVE |
| ILMN_1789418_GSTT2 | 0.31 | 6.24E-09 | YES | negative |
| ILMN_1684391_PLOD1 | 291.16 | 7.25E-09 | YES | POSITIVE |
| ILMN_1675617_NT5M | 0.10 | 1.13E-08 | YES | negative |
| ILMN_1799139_PLOD2 | 15.71 | 1.68E-08 | YES | POSITIVE |
| ILMN_1710027_PNMT | 0.36 | 1.79E-08 | YES | negative |
| ILMN_1693789_ALPP | 0.09 | 2.26E-06 | YES | negative |
| ILMN_1680246_MAT2B | 0.11 | 3.12E-06 | YES | negative |
| ILMN_1786046_CASP9 | 0.41 | 3.36E-04 | YES | negative |
| ILMN_1790680_PDE6D | 17.72 | 5.50E-04 | YES | POSITIVE |
| ILMN_1755974_ALDOC | 1.89 | 1.24E-03 | YES | POSITIVE |
| ILMN_1718070_CASP9 | 0.44 | 2.41E-03 | YES | negative |
| ILMN_1774281_NT5DC3 | 3.68 | 2.36E-02 | YES | POSITIVE |
| ILMN_1673960_MAT2B | 0.55 | 5.90E-02 | no | negative |
| ILMN_2157219_AASS | 0.25 | 9.87E-02 | no | negative |
| ILMN_1771599_PLOD2 | 0.51 | 1.91E-01 | no | negative |
| ILMN_2355549_GSTT2 | 0.71 | 5.46E-01 | no | negative |
| ILMN_1756049_NT5DC3 | 0.84 | 6.24E-01 | no | negative |
